# Barley callus: a model system for bioengineering of starch in cereals

**DOI:** 10.1186/1746-4811-8-36

**Published:** 2012-09-07

**Authors:** Massimiliano Carciofi, Andreas Blennow, Morten M Nielsen, Preben B Holm, Kim H Hebelstrup

**Affiliations:** 1 Department of Molecular Biology and Genetics, Section of Crop Genetics and Biotechnology, Aarhus University, Aarhus, Denmark; 2 Enzymology Group Carlsberg Laboratory, Copenhagen, Denmark; 3 VKR Research Centre for Pro-Active Plants, Department of Plant Biology and Biotechnology, Faculty of Life Sciences, University of Copenhagen, Frederiksberg, Denmark

## Abstract

**Background:**

Starch is the most important source of calories for human nutrition and the majority of it is produced by cereal farming. Starch is also used as a renewable raw material in a range of industrial sectors. It can be chemically modified to introduce new physicochemical properties. In this way starch is adapted to a variety of specific end-uses. Recombinant DNA technologies offers an alternative to starch industrial processing. The plant biosynthetic pathway can be manipulated to design starches with novel structure and improved technological properties. In the future this may reduce or eliminate the economical and environmental costs of industrial modification. Recently, many advances have been achieved to clarify the genetic mechanism that controls starch biosynthesis. Several genes involved in the synthesis and modification of complex carbohydrates in many organisms have been identified and cloned. This knowledge suggests a number of strategies and a series of candidate genes for genetic transformation of crops to generate new types of starch-based polymers. However transformation of cereals is a slow process and there is no easy model system available to test the efficiency of candidate genes *in planta*.

**Results:**

We explored the possibility to use transgenic barley callus generated from immature embryo for a fast test of transgenic modification strategies of starch biosynthesis. We found that this callus contains 4% (w/w dw) starch granules, which we could modify by generating fully transgenic calli by *Agrobacterium*-transformation. A Green Fluorescent Protein reporter protein tag was used to identify and propagate only fully transgenic callus explants. Around 1 – 1.5 g dry weight of fully transgenic callus could be produced in 9 weeks. Callus starch granules were smaller than endosperm starch granules and contained less amylose. Similarly the expression profile of starch biosynthesis genes were slightly different in callus compared with developing endosperm.

**Conclusions:**

In this study we have developed an easy and rapid *in planta* model system for starch bioengineering in cereals. We suggest that this method can be used as a time-efficient model system for fast screening of candidate genes for the generation of modified starch or new types of carbohydrate polymers.

## Background

Starch is a product of plant photosynthetic carbon fixation and is the principal source of energy for human nutrition. Cereal crops produce the majority of starch in agriculture. Starch is also a useful biopolymer used in different industrial sectors [1]. Different crops synthesise structurally and chemically diverse starches with specific physicochemical properties that are suitable for diverse end-uses. However the natural variation found in natural starches does not provide enough different functionalities for the many end-uses. Therefore, after extraction, starch is often processed through a series of chemical or enzymatic modification and physical treatments in order to confer additional properties. Therefore the development of new crops with novel starches, avoiding post-harvest processing, is of economic interest. Genetic modification of the plant starch biosynthetic pathway to design a wide range of tailor made carbohydrates has shown to be a promising strategy [2-6]. The elucidation of the starch biosynthetic mechanism and the characterization of a number of genes, from different origins, involved in polysaccharide modification are offering a rich toolbox for *in planta* production of improved polymers by transgenic technology [7,8]. Therefore it is expected that many novel starches or heterogeneous polysaccharides can be engineered in relevant crops, such as cereals. A severe limitation in the process of testing candidate transgenes is the time required to do genetic transformation, regeneration of transformed plants and characterization of the engineered starch stored in the sink organs (e.g. cereal grains). In barley, which is an important crop and a genetically well characterized cereal model, this time is at least 6 months.

A fast model system for genetic transformation of starch would therefore be useful. The model system should permit the high-throughput screening of candidate transgene activities and the preliminary characterization of the generated modified polysaccharide in a time-efficient way. This could allow to test the validity of several potential transformation strategies. The promising transgenic constructs would be selected to be employed in a cycle of plant transformation, full regeneration and propagation. Such *in vitro* approach may potentially boost the production of new valuable crops synthesizing rationally designed polymers.

Callus tissues of different plants accumulate starch granules especially when they are grown in high sugar media and specific culture conditions, such as controlled temperature, correct hormone treatment and osmotic potential [9-15]. It is not clear why starch is deposited in callus. But it has been suggested that the starch function as an energy reserve for the energy-requiring process of organogenesis (e.g. shoot formation). Alternatively it may play a role in equilibrating the level of free soluble sugars to counterbalance the osmotic potential in the medium [16-20].

In this study we explore the possibility to use transgenic callus of immature barley embryos as a fast model system to study starch biosynthesis and bioengineering of starch in cereals. We optimized a protocol to purify starch from callus. Transgenic callus induced from immature barley embryo after *Agrobacterium*-mediated transformation were found to store significant amounts of starch granules, which are comparable to the ones accumulated in barley storage organs. The system was tested by over-expressing the gene encoding granular bound starch synthase Ia (GBSSIa) tagged with green fluorescent protein (GFP). GBSS is known to synthesize amylose. The transgenic protein localized in the starch granules of the callus and increased the content of amylose.

## Results and Discussion

### Establishing a protocol for generation of transgenic callus to produce starch

**Figure 1:**
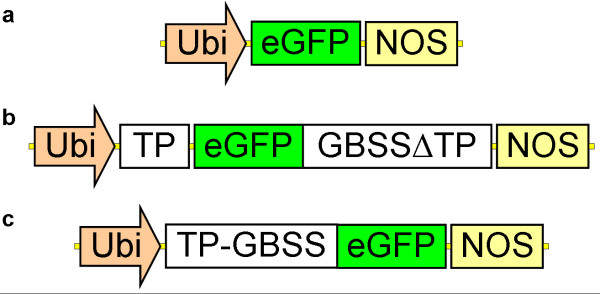
**Transgenic constructs. **pUCE_Ubi:GFP-NOS_, pUCE_Ubi:TP-GFP-HvGBSSIaΔTP:NOS _and pUCE_Ubi:HvGBSSIa-GFP:NOS _constructs. **(a) **The control vector (pUCE_Ubi:GFP:NOS_) was engineered for constitutive expression of the reporter gene encoding enhanced Green Fluorescent Protein (eGFP). **(b) **The pUCE_Ubi:TP-GFP-HvGBSSIaΔTP:NOS _and the **(c) **pUCE_Ubi:HvGBSSIa-GFP:NOS _were designed to overexpress the endogenous barley granular bound starch synthase Ia. HvGBSSIa was fused with GFP. In (b) the GFP was placed after the transit peptide (TP) in GBSSIa. For all constructs, expression was driven by the common maize Ubiquitin promoter. The actual lengths of the individual elements are not drawn to scale.

**Figure 2:**
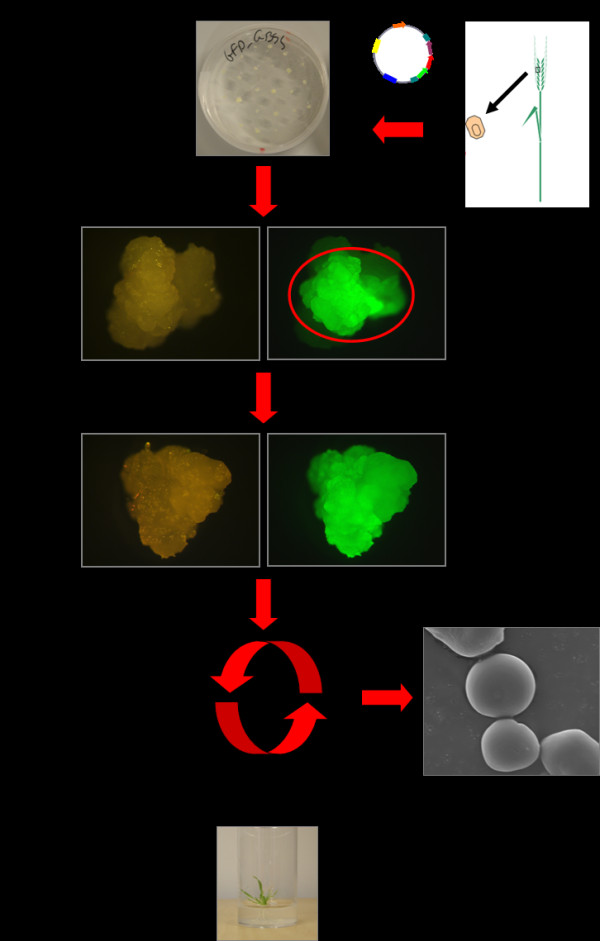
**Schematic workflow of the callus starch models system. **The scheme presents the essential steps. **(a) **Immature embryo transformation. **(b) **Callus containing both transgenic and non-trangenic parts. Transgenic parts are identified by GFP, excised and sub-cultured **(c) **Fully transgenic callus is obtained. **(d) **Callus cultures maintenance with subculturing steps every three weeks. Sampling and starch analysis. **(e) **Transgenic plants can be regenerated.

Three transgenic callus cultures, Ubi:GFP (control), Ubi:GFP:GBSS and Ubi:GBSS:GFP, were obtained from barley immature embryo transformation with the transgenic constructs pUCE_Ubi:GFP-NOS_, pUCE_Ubi:TP-GFP-HvGBSSIaΔTP:NOS_ and pUCE_Ubi:HvGBSSIa-GFP:NOS_ respectively (Figure 1). The pUCE_Ubi:GFP-NOS_ construct which enables constitutive expression of the green fluorescent protein (GFP) reporter gene in the cytosol of the transgenic calli was used as a control to generate transgenic calli without altered GBSS expression. The constructs pUCE_Ubi:TP-GFP-HvGBSSIaΔTP:NOS_ and pUCE_Ubi:HvGBSSIa-GFP:NOS_ were used to over-express GBSSIa tagged with GFP at the N- or C- terminal end, respectively. The presence of a GFP tag allows to identify the cells in each callus, which are expressing the transgenic protein, but is also useful for examination of the sub-cellular localization of the transgenic GBSSIa enzyme. In addition to the GFP marker, a hygromycin selection marker was used to select for growth of transgenic cells over non-transgenic cells. One hundred and fifty embryos were transfected for each of the three constructs. After the first three weeks on selective medium the calli consisted of a mosaic of transgenic/fluorescent and non-transgenic/non-fluorescent cells. Those parts of callus tissues not showing fluorescence were removed by excision. The remaining pieces of calli were sub-cultured into the medium of new petri dishes for additionally 3 weeks, after which the calli consisted only of transgenic cells. After another additional sub-culturing period most of the calli were pooled from each of the lines and harvested. Between 1 and 1.5 g dw tissue was obtained for each of the three different constructs, from which we could purify between 5 and 10 mg of starch from each. However we suggest that it may be possible to optimize culture conditions (e.g. hormones concentration, temperature, carbohydrate concentration) in order to increase starch content, influence the starch biosynthetic activity pathway and alter starch composition [9,11,12,21,22]. This could increase the resemblance among the callus and the endosperm system, increasing the in vitro culture ability to serve as a model system [23]. The starch is purified from a pool of calli, which originate from different transformation events, whereby the expression level of the enzyme is an average of multiple T-DNA insertion events, After characterization of the callus starch, we were able to regenerate plants from the remaining calli. The complete strategy is schematized in Figure 2.

### Characterization of starch granules in barley callus

**Figure 3:**
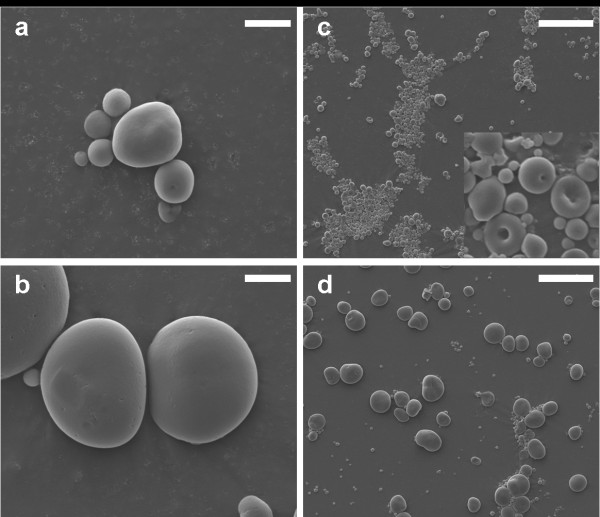
**Scanning electron microscopy (SEM) of starch granules. **Scanning electron microscopy (SEM) pictures of **(a,c) **Ubi:GFP control callus and **(b,d) **barley endosperm starch granules. A magnification of starch granules with the characteristic doughnut-shape is indicated in the lower right corner of (c). Scale bars represent 50 μm in **(c,d) **and 5 μm in **(a,b).**

Calli were cut in 30 μm thick sections with a microtome. Presence of starch granules in the control callus tissues was observed with light microscopy after staining with Lugol iodine. The staining showed the presence of numerous starch granules deposited inside cells (Additional file 1: Figure S1). This confirmed previous studies, which indicated that barley callus contain starch granules [13,24,25]. The starch content of the barley callus at the time of harvesting was determined as described in materials and methods. We found that the callus contained 4.4% starch (St. Dev ± 0.4, n = 7) of the dry weight. This value is less than that of developing barley grains (15–20 DAP), which was determined to be 35.4% (St. Dev ± 6.0, n = 7). However it is comparable to the starch content in the early developmental stages of the grain [26,27]. Similar amounts of starch were also found in potato and sweet potato callus tissue, and in suspension cell cultures of rice [9,10,21,23,28]. As reported above, the actual amount of starch which could be purified from callus was 5 – 10 mg per 1 g dw (0.5–1% w/w), which suggests that more than 75% of the starch is lost during the purification process. Starch granule morphology was studied by scanning electron microscopy. Starch granules isolated from callus tissues were round with a smooth surface resembling granules purified form barley endosperm except that they were smaller (Diameters between 2 and 10 μm, Figure 3). A subpopulation of starch granules exhibited a peculiar “doughnut-shaped” morphology, which is unusual for barley starch granules Such collapsed morphology was reported in starch of some sorghum genotypes and is associated with a more digestible starch type [29,30].

**Figure 4:**
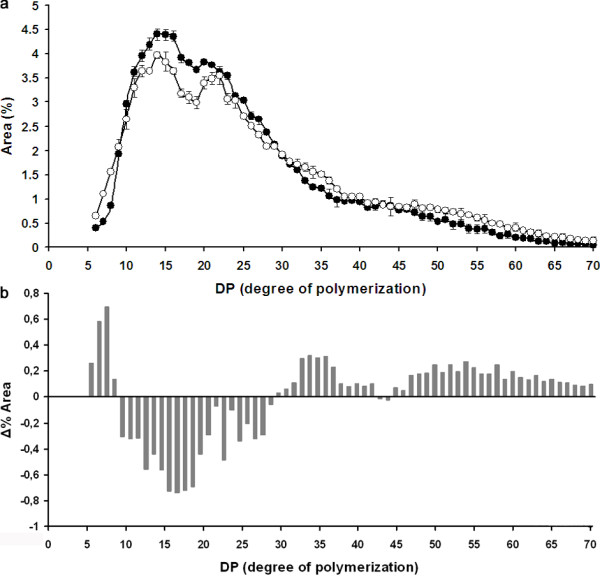
**Profile of chain length distributions. **Chain length distribution **(a) **of amylopectin isolated from Ubi:GFP callus (white dots) and barley endosperm (black dots). The numbers are based on duplicates. Bars represent standard deviations. **(b) **Relative differences in the frequency of chain lengths between amylopectin isolated from Ubi:GFP control callus and barley endosperm.

The callus starch was further examined by using high performance anion-exchange chromatography with pulsed amperometric detection to determine the distribution of chain length in de-branched amylopectin. The chain length distribution was compared with endosperm starch (Figure 4). Callus and endosperm starch both have a bimodal distribution of the chain lengths peaking at 14 DP (degree of polymerization) for peak I. However peak II is at 20 and 22 DP for amylopectin from barley endosperm and callus respectively. In addition, callus starch has a higher proportions of the very short chains (DP 6–9), long chains (DP 33–39) and very long chains (> DP 50). In contrast starch from barley endosperm has a higher amount in short and intermediate chains (DP 10–28).

### Expression of genes involved in starch biosynthesis in callus

The major starch storage tissue in cereal plants is the filial starchy endosperm. However other tissues in the seed and in other organs accumulate starch in a transitory form, such as the aleurone, embryo, pericarp and leaf tissues [26]. The starch is shaped differently in the various tissues due to differential expression of genes, coding for starch biosynthesis enzymes and other factors involved in the shaping of starch [26].

**Figure 5:**
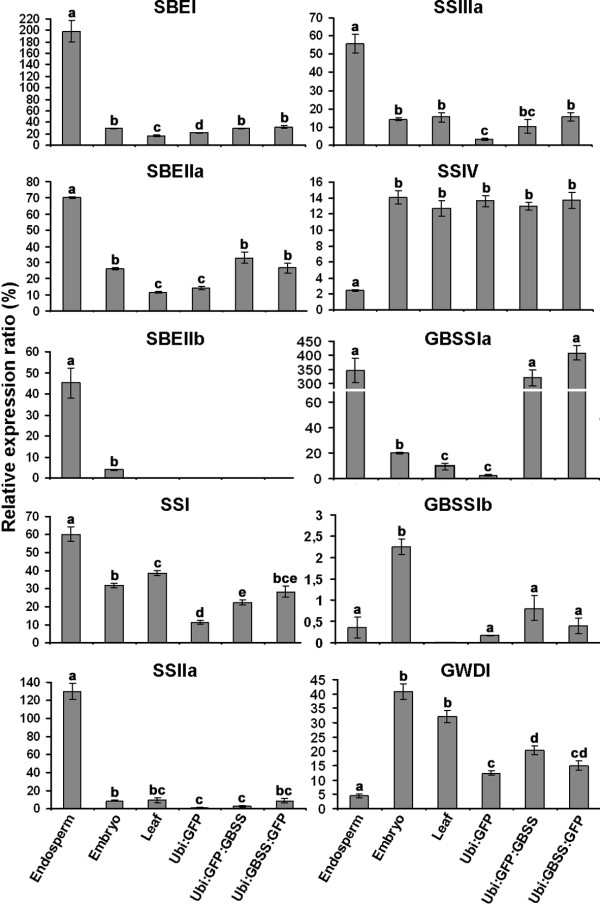
**Expression patterns. **Transcript profiles of starch biosynthetic enzymes in endosperm, embryo, leaf, Ubi:GFP callus, Ubi:GFP:GBSS callus and Ubi:GBSS:GFP callus. Bars indicate standard deviations. Letters above bars indicate statistical difference at P < 0.05.

We studied the gene expression profiles of genes coding for selected enzymes involved in the synthesis and shaping of starch callus tissues and compared with barley leaf, 15–20 DAP developing endosperm and embryo: starch branching enzyme I (SBEI), starch branching enzyme IIa (SBE IIa), starch synthase I (SSI), starch synthase IIa (SSIIa), starch synthase IIIa (SSIIIa), starch synthase IV (SSIV), granule bound starch synthase Ia (GBSSIa), granule bound starch synthase Ib (GBSSIb), glucan water dikinase I (GWD) (Figure 5). Expression levels were estimated by qRT-PCR. The aim was to test whether the expression profile of the genes associated with starch biosynthesis in barley calli was comparable with the one in barley endosperm or if it has more resemblance with the expression profile of a transitory storage organ (such as leaf or embryo). Most of the tested genes involved in starch biosynthesis are expressed in callus, with exception of starch-branching enzyme IIb (SBE IIb), which was neither expressed in callus nor in leaves. In general the level of expression of the starch synthases were lower in callus than in endosperm, which is well in line with the observation, that callus contains less starch than the endosperm does. Starch synthase IV was an exception to this, with a significant higher expression in callus (and embryos and leaves) than in endosperm. Studies in *Arabidopsis thaliana* have indicated that starch synthase IV type enzymes play a role in the control of starch granule numbers and size, possible by priming starch synthesis, because plants without SS IV contain less but bigger starch granules and same amount of starch [31]. A higher SS IV gene expression level in callus as compared to endosperm is therefore consistent with the observation that callus contains smaller granules than endosperm does. Overall, the pattern of gene expression in callus has more similarity with the pattern detected in embryos and leaves. The biosynthesis of transient starch in chloroplasts in leaves is essentially similar to the biosynthesis of storage starch in amyloplasts [32]. In both tissues glucose is utilized from ADP-glucose by starch synthases to elongate starch chains. However the difference among the tissues is believed to be due to differential concentrations of the various forms of starch synthases, which have different chain length preferences [32]. The observation that there is a difference in chain length distribution between callus and endosperm starch (Figure 4) is therefore well in line with our observation that the two tissues have different expression patterns of starch synthases. For example GBSSIa and SSIIa gene expression is barely detectable in callus. However, this implies that the callus system would be particularly well suited for studying the effect of expressing different variants of these two starch synthases, because only little is expressed already. Interestingly it has been observed in rice breeding populations that polymorphism in the GBSSIa and SSIIa genes is responsible for the majority of variation in gelatinization temperature and other functionalities of starch [33,34].

### Overexpression of GFP tagged GBSS in callus: proof-of-concept

As a proof-of-concept, that starch callus can be engineered by transgenic techniques we overexpressed GBSSIa tagged with GFP (Ubi:GBSS-GFP and Ubi:GFP-GBSS) in callus as described above. We observed that control callus (Ubi:GFP) has a very low gene expression level of GBSSI (Figure 5), which suggest that it contains less amylose than endosperm starch does. This was confirmed by direct measurement of amylose content in starch purified from endosperm and control callus (Ubi:GFP) respectively, which showed that the callus starch only contains 2.2% amylose, whereas endosperm starch from the same barley variety contains 24.5% amylose (Table 1). Overexpression of GBSSIa-GFP or GFP-GBSSIa was confirmed by qRT-PCR studies, which demonstrated that Ubi:GFP:GBSS and Ubi:GBSS:GFP calli had an up-regulation in gene expression of GBSSIa at 126.5 and 160.7 folds respectively, compared with control callus (Ubi:GFP). This level of GBSSIa gene expression in Ubi:GBSS-GFP and Ubi:GFP-GBSS were similar to that of developing endosperm (Figure 5). The overexpression of GBSSIa increased the amount of amylose in callus starch from 2.2% in the control callus to 11.7% in Ubi:GFP:GBSS and 27.9% in Ubi:GBSS:GFP callus starches (Table 1). For Ubi:GBSS-GFP this level is in the range of that of developing endosperm (24.5%). This demonstrates by an example that we are able to modify starch biosynthesis in callus starch by transgenically changing gene expression. GBSSIa over-expression had an effect on the expression of some of the other genes involved in starch biosynthesis. The expression level of the SBEI, SBEIIa and SSI genes were all increased in the Ubi:GBSS-GFP and Ubi:GFP-GBSS calli compared with control callus (Ubi:GFP).

**Table 1:** Amylose content

**Sample**	**Amylose content (%) ± SD**
Barley endosperm	24.5 ± 0.8
Callus Ubi:GFP (control)	2.2 ± 0.2
Callus Ubi:GBSS:GFP	27.9 ± 0.7
Callus Ubi:GFP:GBSS	11.7 ± 1.2

Amylose content analysis (average values of 2 biological and 6 technical replicates are indicated).

**Figure 6:**
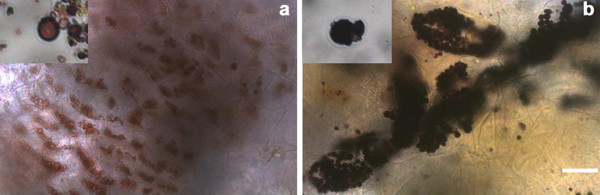
**Morphology of control and transgenic callus starch. **Microscopy of callus and purified starch stained with Lugol iodine **(a) **Ubi:GFP mashed tissue (lower magnification) and purified granules (higher magnification, upper left corner). **(b) **Ubi:GBSS:GFP mashed tissue (lower magnification) and purified granules (higher magnification, upper left corner). Scale bar represents 40 μm for lower magnification.

Before the extraction process, samples of calli were stained with Lugol iodine stain solution and examined by microscopy. Starch granules deposited in Ubi:GBSS: GFP tissues stained dark blue in contrast to the brownish granules found in Ubi:GFP calli, confirming that these granules contain a higher amount of amylose (Figure 6). The same color difference was observed in purified granules. We did not observe any difference in the shape or size of Ubi:GBSS-GFP and control (Ubi:GFP) starch granules, either in the callus or in the purified starch fraction.

Polymorphism in the genes encoding starch synthases have been demonstrated to be associated with variation in starch functionality such as gelatinization temperature and retrogradation [33,34]. In addition wild relatives of cereals contain a significantly higher diversity of genes encoding enzymes involved in starch biosynthesis than domesticated species. Wild cereals may therefore contribute to the breeding of cereal varieties with new starch functionalities [35]. However, association studies cannot directly demonstrate the effect of amino acid sequence variation in enzymes on starch functionality. We suggest that the callus starch model presented here can be used as a fast screening method to directly evaluate the impact of such known variants of starch synthases in gene pools of breeding populations and wild cereal species.

### Binding of GFP tagged GBSS in starch granules

**Figure 7:**
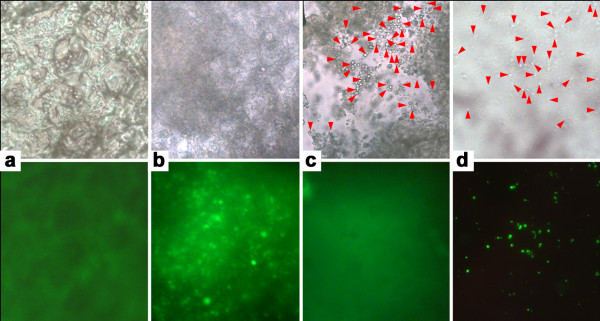
**Fluorescence microscopy. **Bright field (upper figures) and fluorescence (lower figures) microscopy. **(a) **Intact Ubi:GFP callus. **(b) **Intact Ubi:GFP:GBSS callus. **(c) **30 μm section of mashed Ubi:GFP callus. **(d) **30 μm section of mashed Ubi:GFP:GBSS callus. Ubi:GFP:GBSS callus shows a fluorescence pattern localized in small spots (b) and in the starch granules (d). The red arrows point at starch granules.

The use of a GFP tag on the overexpressed enzyme allowed us to visualize its subcellular localization. In control callus (Ubi:GFP) GFP was found in the cytosol (Figure 7a) whereas in tissues of transgenic callus transformed with Ubi:GFP-GBSS construct the fluorescence was localized in small spots within the cells (Figure 7b). When the calli were cut in 30 μm it was possible to clearly distinguish starch granules within debris from smashed cells, in both control and Ubi:GFP:GBSS samples (Figure 7 c,d – upper panels). In control Ubi:GFP callus, GFP was floating around in the cell debris giving a general high background without specific localization (Figure 7c – lower panel). While in tissue sections of Ubi:GFP:GBSS callus the GFP was found into the starch granules (Figure 7d – lower panel). These results were in agreement with our expectation, that the fused Ubi:GFP:GBSS protein was targeted into the plastids and localized within starch granules. Furthermore, they demonstrate that the callus starch model system can be used to visualize the binding of GBSSIa in starch granules.

## Conclusions

Plant callus tissues have been used as a tool for biochemical studies of the parent tissues [23,36]. Attempts have been conducted to validate callus and suspension cell cultures as model systems to investigate carbohydrate and starch metabolism in storage organs of different plants, such as maize, rice, potato and sweet potato [10,21-23,28,37].

In our study we aimed to demonstrate that transgenic barley callus may represent a valid model system for starch bioengineering by transgenic transformation of the callus.

We analysed the starch produced by callus generated from immature barley embryos. There is a lower quantity of starch in callus than in endosperm tissue. The starch granules are smaller and contain only little amylose. We examined the expression profile of various genes involved in starch biosynthesis. Callus has a different expression profile than endosperm of the various forms of starch synthases, which is reflected in a different chain length distribution of the starch. We suggest that among the starch synthases the callus system may be particularly well suited for studying the effect of expression of transgenic variants of GBSSIa and SSIa, because these two genes only have very little expression in callus already. We used GFP as a tag for expression of transgenic enzymes in the callus. GFP fluorescence was easily detectable in the callus, and could be used to validate gene transfer to the callus and localize transgenic cells. Fluorescent transgenic cells could be isolated by cutting the callus with a scalpel and sub-culture it to obtain stable transgenic callus (Figure 2). 1 g of dry callus could be obtained after 9 weeks of growth, from which we were able to purify > 5 mg starch. By using GBSSIa as an example we demonstrated that this method could be applied to overexpress genes of enzymes in callus to engineer starch biosynthesis. The transgenic callus had a significantly increased content of amylose, which is expected if the enzyme is active in the callus. In addition we were able to visualize the physical binding of GBSSIa in starch granules. In summary we suggest that the method can be used to do fast in vivo screening of variants of genes encoding enzymes involved in starch biosynthesis or other potential transgenes for bioengineering of starch in cereals. We also suggest that the method can be used to study the binding of proteins such as starch synthases in starch granules.

## Methods

### Vector engineering and transgenic callus generation

Transgenic callus cultures were induced from immature embryos of Golden Promise, by *Agrobacterium*-mediated transformation as described previously [38]. Three plasmid vectors were used for transformation: a control vector (pUCE_Ubi:GFP-NOS_) and two vectors designed to manipulate starch synthesis altering amylose content (pUCE_Ubi:TP-GFP-HvGBSSIaΔTP:NOS_ and pUCE_Ubi:HvGBSSIa-GFP:NOS_). They were engineered by using the pUCE tool box for construction of cereal transformation vectors as described previously [39]. They all contain the Hygromycin phosphotransferase selection marker gene (HPT), and the common maize ubiquitin promoter for constitutive expression of the transgene. In addition green fluorescent protein (GFP) was used as a marker. The control vector (pUCE_Ubi:GFP:NOS_) was engineered for constitutive expression of GFP without an enzyme . The other binary vectors were designed to overexpress barley granule bound starch synthase Ia. (HvGBSSIa), In the case of pUCE_Ubi:TP-GFP-HvGBSSIaΔTP:NOS_ the transit peptide (TP) from GBSSIa was attached in front of GFP. The GBSSI-GFP and GFP-GBSSI sequences were synthesized artificially by DNA2.0 (http://www.dna20.com) fusing together the coding sequence of GFP with the coding sequence of HvGBSS with or without the native transit peptide, respectively. The two constructs were provided by the company in their cloning vectors and they were both digested with *PacI* (Fermentas, FastDigest), and re-cloned into the pUCE vectors Ubi:USER:NOS and Ubi:TP-USER:NOS (*PacI* digested and treated with calf intestine alkaline phosphatase) respectively. The two binary vectors are described in details in [39].

### Transgenic callus maintenance and culture procedures

After two days on callus induction media the embryos were transferred to callus production media as described in the protocol in [38]. After three weeks of culturing on production media the calli were examined with a ‘Wild MZ8 Leica’ stereo microscope, equipped with a GFP fluorescence detection system and calli pieces of full transgenic tissues were excised and sub-cultured on fresh production media to obtain homogeneous fully transgenic calli. Callus cultures were sub-cultured one additional time after 3 weeks, and after 9 weeks total the transgenic calli tissues were harvested and snap-freezed in liquid nitrogen and stored in a freezer (−80) to be directly used for analysis or for starch granules purification.

### Light microscopy observations

For light microscopy analysis, 30 μm sections were cut from fresh frozen callus tissues using a HM 550 OM Cryostat microtome. Callus tissue sections, mashed tissue and purified starch granules were stained with Lugol´s iodine stain solution (250 mg I_2_, 2.5 g KI, 125 mL ddH_2_O) and mounted on a Zeiss Axioplan 2 Imaging microscope. Pictures were taken using the software provided by the manufacturer.

### Measurement of starch content

Callus tissue was freeze-dried using a Heto LyoPro 6000 freeze dryer and ground using mortar and pestle. Pulverized material was used for starch content analysis. Starch content was determined using the ‘Total starch AOAC Method 996.11/AACC Method 76.13’ kit from Megazyme International Ltd. (Wicklow, Ireland) using the protocol recommended by the manufacturer for samples containing also d-glucose and/or maltodextrins.

### Extraction of total RNA and quantitative real-time PCR

Total RNA was purified from transgenic callus tissues, barley endosperm 15–20 DAP and barley leaves; and cDNA was synthesised and quantitative real-time PCR (RT qPCR was conducted as described in [38]. Glyceraldehyde-3-phosphate dehydrogenase (*GAPDH*) was co-amplified as housekeeping gene to calculate relative quantification of expression [40]. All the analyses were conducted in three biological replicates each with three technical replicates using specific primers targeting the following starch biosynthetic genes: starch branching enzyme I (*SbeI*), starch branching enzyme IIa (*SbeIIa*), starch branching enzyme IIb (*SbeIIb*), starch synthase I (*SSI*), starch synthase IIa (*SSIIa*), starch synthase IIIa (*SSIIIa*), starch synthase IV (*SSIV*), granule bound starch synthase Ia (*GBSSIa*), granule bound starch synthase Ib (*GBSSIb*), glucan water dikinase I (*GWDI*). The primer sequences are described in Additional file 2: Table S1. Student’s t-test was used to compare the levels of expression of the same genes among the different tissues (with 95% confidence level).

### Starch extraction and purification

Starch purification form barley grain was conducted according to the protocol described in Additional file 3: Protocol.

### Scanning electron microscopy

Scanning electron microscopy was carried out as described in [38].

### Amylose determination

The amylose content was determined by iodine colorimetrics following the method described in [41].

### Chain-length distribution of starch

Chain length distribution was assessed according to the protocol described in [42,43].

## Competing interests

The authors declare that they have no competing interests.

## Authors’ contributions

MC developed protocols for purification of callus starch and carried out the starch and gene expression analysis and participated with a major contribution to construction of vectors and callus transformation and with a major contribution to the writing of the manuscript. AB supervised, coordinated and participated in the starch analysis and analyzing of data. MMN participated in the construction of vectors. PBH supervised on transformation and genetics of barley. KHH conceived of the study and coordinated the design and analysis of experiments, and participated with a major contribution in the construction of vectors, transformation of callus and fluorescence microscopy and participated with a major contribution to the writing and editing of the manuscript. All authors read and approved the final manuscript.

## Supplementary Material

Additional file 1 **Figure S1. **Lugol iodine stain of callus. Click here for file

Additional file 2 **Table S1. **Primers. Click here for file

Additional file 3 Protocol. Click here for file
