# Supplementary material for: Barley callus: a model system for bioengineering of starch in cereals
Source: Plant Methods. 2012 Sep 7;8:36. doi: 10.1186/1746-4811-8-36 (PMC3479045; doi:10.1186/1746-4811-8-36)

Supplementary figure 1 - Lugol iodine stain of callus

Ubi:GFP callus cells containing starch granules. The bar equals 25 µm.

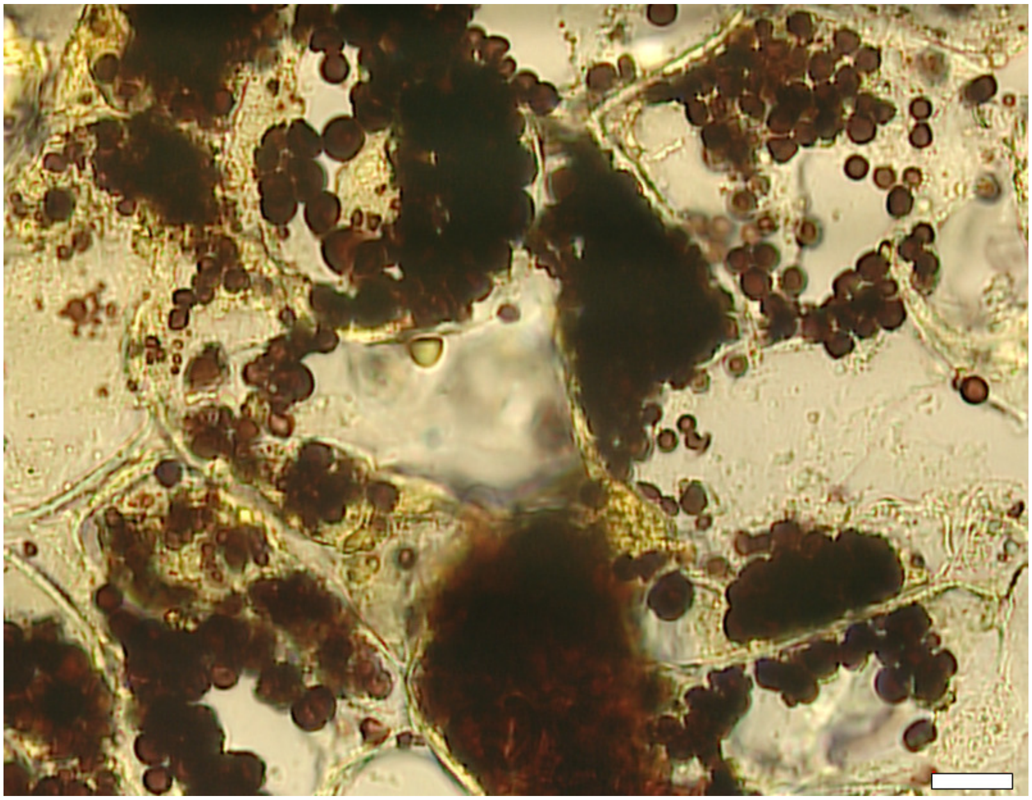

Supplement: Additional file 1 — Figure S1. Lugol iodine stain of callus. [file 1746-4811-8-36-S1.pdf]
