# Supplementary material for: Barley callus: a model system for bioengineering of starch in cereals
Source: Plant Methods. 2012 Sep 7;8:36. doi: 10.1186/1746-4811-8-36 (PMC3479045; doi:10.1186/1746-4811-8-36)
Supplement: Additional file 2 — Table S1. Primers. [file 1746-4811-8-36-S2.pdf]

Primer Name	Sequence
GAPDH Fw	5´-GCTCAAGGGTATCATGGGTTACG-3´
GAPDH Rev	5´-GCAATTCCACCCTTAGCATCAAAG-3´
Sbe I Fw	5´-TGATTGACGAACACGAGGGA-3´
Sbe I Rev	5´-TCCCGTTGACATGGGAAATC-3´
Sbe IIa Fw	5´-GAGCCATCTTGACTACCGAT-3´
Sbe IIa Rev	5´-GGGAGGAAAATCTCCCAAAC-3´
Sbe IIb Fw	5´-AAGCGGAACACCGCCTTC-3´
Sbe IIb Rev	5´-GGTTGTGGCACAATGCGTAT-3´
SSI Fw	5´-TCGAAGGGATTGCTGAGGAT-3´
SSI Rev	5´-AGCAGCAAGAGCAATTGGCA-3´
SSIIa Fw	5´-CCGCTGTACAAGAAGACCTT-3´
SSIIa Rev	5´-CAAGACCACCTGTTTTGCAC-3´
SSIIIa Fw	5´-TGAAAAGAAAGGCTGAGAGAAG-3´
SSIIIa Rev	5´-AGGAGCATCTAAACCAACCC-3´
SSIV Fw	5´-CTGCATTTGTTGCACCTCTTTA-3´
SSIV Rev	5´-CAACTGCACCCTTAACAGCA-3´
GBSSIa Fw	5´-TCATCTCCGAGATCAAGGTC-3´
GBSSIa Rev	5´-GAGGTTGAGGATCCTGGG-3´
GBSSIb Fw	5´-CGGCACAGGGAAGAAGAAAA-3´
GBSSIb Rev	5´-GATGGGACCAACTCCATAGC-3´
GWDI Fw	5´-AATTCGTGGTGGATCAGCTG-3´
GWDI Rev	5´-CACCTTGCAATTCCTTGCTC-3´
