# Supplementary material for: Barley callus: a model system for bioengineering of starch in cereals
Source: Plant Methods. 2012 Sep 7;8:36. doi: 10.1186/1746-4811-8-36 (PMC3479045; doi:10.1186/1746-4811-8-36)
Supplement: Additional file 3 — Protocol. [file 1746-4811-8-36-S3.pdf]

Extraction of Starch from barley embryonic callus

Massimiliano Carciofi , Aarhus University
&
Andreas Blennow, Copenhagen University

Updated: June 2012

1. Freeze dry the callus tissue

2. Grind the callus tissue (~1 g) into a fine powder with mortar and pestle

3. Add approx 50 ml of 5 mM DTT (dithiotreitol) in 1% SDS (sodium dodecyl sulphate) and mix vigorously (you can help suspending by regularly pipetting the slurry with plastic Pasteur pipettes while adding 5 mM DTT in 1% SDS).

4. Incubate for 30 min while mixing (use a shaker).

5. Centrifuge at 5500 g for 10 min. Discard the supernatant.

6. Repeat step 2-4 twice.

7. Add approx 50 ml of ddH_2O and resuspend the pellet (regularly pipetting with a plastic Pasteur pipette)

8. Centrifuge at 5500 g for 10 min. Discard the supernatant.

9. Repeat steps 6 and 7 twice.

10. Sieve the slurry through a 35 μm sieve and collect the flow through in a 50 ml Falcon tube. Keep adding distilled water to the slurry until there are no granules anymore in the flow through (This requires around 200 ml of ddH_2O corresponding to 4 Falcons tube for each sample). Centrifuge at 5500 g for 10 min, discard the supernatant leaving a small volume of water (~5 ml).

11. Resuspend the starch in the small volume of water and transfer all the starch from the 4 Falcon tubes to one of them (wash the sides of the 4 Falcon tubes with a few ml of ddH_2O to be sure not to lose any starch) reaching a final volume of 50 ml. The final result will be one Falcon tube containing all the starch from one sample resuspended in 50 ml.

12. Allow to sediment over 2 days at 4º C (or centrifuge at 5500 g for 30 min) and discard the supernatant leaving a small volume of water (~5 ml). Resuspend the sediment in the small volume of water.

Note: Make sure to keep checking in a microscope if you are loosing starch granules.

13. Then subject to a density gradient centrifugation using either with CsCl (80 %w/v) or 6M NaCl / 50 % sucrose solution (105g NaCl, 300g Sucrose, 300 ml water).
Procedure: The total sediment from each callus sample is resuspended in aliquots of 1 ml, which should be carefully layered above 0.8 mL of CsCl or NaCl / Sucrose solution (inside a 2 ml eppendorf tube) and centrifuged at 14,000 g for 7 minutes.

14. The supernatant is then discarded and the sediment of starch is washed 3 times in ddH_2O (i.e. you suspend the pellet in water and centrifuge at 14,000g each time)

15. Add approx 1 ml of ethanol 96% and resuspend by gently pipetting. Centrifuge at 5500 g for 10 min. Discard the supernatant. Repeat it twice.

16. Dry the pellet at room temperature.

Remarks:
Centrifugation: check that everything is pelleted. Small starch granules are sometimes hard to pellet. You can adjust the speed of the centrifuge if necessary. The pellet can be too hard if centrifugation is too fast. It is important to completely resuspend the pellet.
